# Agarose Gel: An Overview of the Dermal Filler and a Clinical Experience With 700 Patients

**DOI:** 10.1093/asjof/ojad051

**Published:** 2023-06-14

**Authors:** Omer Buhsem, Ahmet Kirazoglu

## Abstract

**Background:**

Dermal fillers currently in the market have several advantages and disadvantages over each other. Agarose gel (AG) is a unique material due to its special rheological characteristics and gel-forming capability.

**Objectives:**

The authors aimed to share their clinical experience on AG for a variety of facial augmentation procedures and its long-term results.

**Methods:**

The study population consisted of 700 patients (532 females; 168 males) aged 18 to 52 years. Follow-up visits were at 1, 3, 6, 12, and 24 months after the injections. Patient satisfaction was evaluated on a scale from 0 to 10 using a survey and clinical improvement was evaluated using the Global Aesthetic Improvement Scale (GAIS) by 2 independent plastic surgeons before the injection and at 1-year follow-up.

**Results:**

Eighty-two percent of the patients scored 1 or 2 (exceptional or great improvement) on GAIS. Eighty-five percent of the patients scored 8 or above (very satisfied). Most patients experienced at least 80% persistence of effect at 1-year follow-up.

**Conclusions:**

AG appears to be suitable for a variety of facial augmentation and contouring applications, as it is safe and has long-lasting favorable cosmetic efficacy.

**Level of Evidence: 3:**

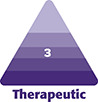

For over a century, a multitude of experimental and clinical research studies in the field of plastic surgery have been dedicated to finding appropriate tissue fillers to correct contour deformities. Presently, filler treatments have emerged as an elegant and highly effective cosmetic solution used widely across the globe.

Fillers, which can be classified according to their various properties, could be categorized as temporary, semi-permanent or permanent according to the product composition or the substance duration in the tissue. Hyaluronic acid (HA), calcium hydroxylapatite, collagen, poly-l-lactic acid, polymethylmethacrylate, and agarose gel (AG) are the most commonly used types in the practice.^1-4^

Filling procedures require appropriate patient selection as well as good anatomical knowledge, correct injection technique, safety, and competence obtained from correctly selected products. As the indications of dermal fillers for the ever-expanding range and the number of procedures performed increase, so will the number of complications that may occur due to foreign body reaction and the presence of nonbiodegradable chemicals.

In recent years, many materials have been used as tissue fillers, but it would not be correct to acknowledge any of them as ideal fillers. It is important to choose the filler according to its characteristics. A desirable material for injectables should meet several criteria. It must be safe and effective, highly biocompatible, and nonimmunogenic, to avoid any significant inflammatory response. In addition, the biomaterial should possess the ability to retain its form, exhibit a suitable rate of biodegradation at the implant site, and demonstrate longevity. Furthermore, it should facilitate the transportation and preservation of cells or signaling molecules at the site of implantation, while exhibiting minimal potential for intratissue migration. The material should also be affordable and easily storable. It is important to assess these fundamental characteristics to ensure utmost safety and efficacy in clinical use.^5-16^

Agarose originates from sea algae. It is a saccharide polymer and dissolves into water forms a gel with a three-dimensional (3D) porous reticulum. AG is viscous-elastic at temperatures below 45 C. It is slowly desorbed by macrophages, and intracellular metabolism through pentose cycle (Figure 1).^17-20^

**Figure 1.**
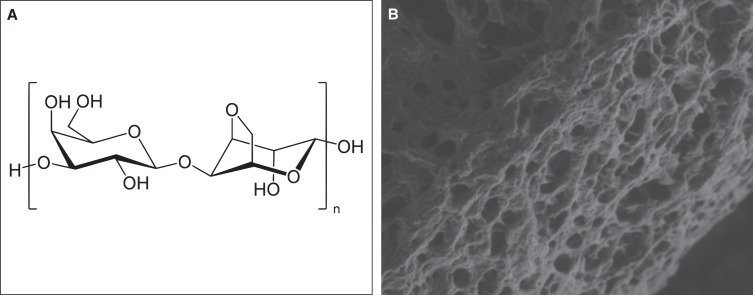
(A) Chemical structure of agarose. (B) The 3D structure of the AG, with pores of different size, observed by scanning electron microscope.

There are many fillers on the market with different properties. AG is an option with significant advantages in certain indications. It is a unique material with gel-forming ability and special rheological properties.^19,21,22^

The important characteristics of this filler to point out are:

- AG is nonhydrophilic and it has high G-prime properties which allow the practitioner for accurate injections. It does not have a swelling effect which may disrupt the cosmetic appearance; so that, it is likely to achieve better contour and definition, making the result of the treatment immediately appreciable.^19,23,24^
- AG does not cause any edema in the surrounding tissues in the mid-term and long-term.^19,23^
- Due to its slow resorption feature, it remains in place for an extended period of time and this positively affects the clinical result.^23^
- Due to its nonreticular molecular structure, the probability of migration is very low.^19,23,24^
- AG is a hydrogel whose consistency is determined by hydrogen bonds between the linear chains. It is nontoxic and it does not contain any reticulating and/or crosslinking agents such as 1,4-butanediol diglycidyl ether or any other chemical agents which are present in HA fillers.^25,26^
- Being natural absorbable filler, it is completely biodegradable. The feature that makes AG safe is that it can be removed from the injection site by macrophage phagocytosis and intracellular metabolism through the pentose cycle.^19,21-25^

AG is currently being used in 45 countries, including Europe. It comes in 4 different concentrations including 1%, 1.5%, 2.5%, and 3.5% agarose in a saline solution. The 2.5% and 3.5% AG fillers also contain 0.4% nonreticulated HA. It is suggested to use higher concentrations for injecting deeper into areas that require more volume, such as the zygoma or chin, while lower concentrations are appropriate for injecting into the more superficial subdermal plane in areas such as marionette or nasolabial lines. Although pain is not seen with slow injection, it is possible to mix with 0.1 to 0.2 mL of 2% lidocaine to prevent the possible discomfort, which means total amount of 1.6 mL of mixture is available. The product is homogenized by transferring it between syringes with Luer-lock fittings, typically 5 to 10 times, until the mixture is uniform.

## METHODS

A variety of facial augmentation applications using AG dermal filler (Algeness Advanced Aesthetic Technologies; Brookline, MA) were performed on patients between February 2020 and February 2022 at our clinic. Patients over the age of 18 were selected to be eligible for inclusion and required that patients have facial soft-tissue deficiency. The study excluded patients who had received permanent implants or previous soft-tissue augmentation, as well as those with a known allergy to fillers, recurring skin disease, history of connective tissue disease, or active infection at the time of the study.

Bezmialem Vakif University institutional review board approved the study protocol. Also, this study adhered to the guidelines of the Declaration of Helsinki. Written consent was obtained from all patients, allowing the use and analysis of their data.

### Areas of Treatment and Procedure

The senior author conducted each procedure in the office. After the injection was administered, the treated area was subjected to gentle massage, and the patients were closely monitored for any adverse reactions. On average, the treatment duration was 20 min.

The primary areas treated were chin, nasolabial area, temple, zygoma, and nose. The most commonly treated site was the zygoma (188 patients). AG was administered with a 23 G blunt tip rigid cannula or 27 G 13 mm needle provided by the manufacturer. The precise technique, injection volumes, and concentrations used varied. Generally, a linear threading, small boluses, and big boluses techniques were used. Injection planes were the supraperiosteal, subdermal, and/or subsuperficial musculoaponeurotic system (SMAS) planes, depending on the area being augmented. Since the migration feature of AG is low, linear threading technique is applied to avoid lump formation especially in soft tissue and subdermal applications (Table 1). Example of injection techniques and instructions can be seen in Videos 1 and 2.

**Table 1.** Areas Treated With AG in Our Study

Areas Treated with AG	Chin	Temple	Zygoma	Nose	Nasolabial area, smile lines
Patient number	179	45	188	116	172
Mean age	24	44	32	23	39
Injection plane	SP	SP	SP	SP/subSMAS	SP/subdermal
Injection technique	Small boluses/LT	Big bolus	LT	Small boluses/LT	Small boluses/LT
Amount of filler (range), cc	1.4-2.8	1.4-2.8	1.4-2.8	0.7-1.4	1.4-4.2
Amount of filler (average), cc	2.1	1 ES	1.1 ES	1	1.4 ES
AG concentration	3.5%	3.5%	3.5%/2.5%	3.5%/2.5%	3.5%/2.5%

AG, agarose gel; ES, each side; LT, linear threading; SMAS, superficial musculoaponeurotic system; SP, supraperiosteal.

### Patient Follow-up and Evaluation

Follow-up visits were at 1, 3, 6, 12, and 24 months after the injections. All patients were followed up >2 years. Standardized digital photography was used and taken before the treatment and at the follow-up visits. To assess patient satisfaction, a survey was conducted. The clinical improvement was evaluated using the Global Aesthetic Improvement Scale (GAIS) by 2 independent plastic surgeons. The assessments were performed before the injection and at a 1-year follow-up (as presented in Table 2). Additionally, patients were monitored after the injection for any complications or the need for revisional “touch-up” procedures. Touch-ups were performed 2 to 4 weeks after the initial procedure.

**Table 2.** Global Aesthetic Improvement Scale Assessment

Global Aesthetic Improvement Scale		
Score	Degree	Description
1	Exceptional improvement	Excellent corrective result
2	Very improved	Marked improvement of the appearance, but not completely optimal
3	Mild improvement	Improved of the appearance, better compared to the initial condition, but a touch-up is advised
4	Unaltered patient	The appearance substantially remains the same compared to the original condition
5	Worsened patient	The appearance has worsened with the original condition

### Statistical Analysis

A descriptive analysis was conducted. Categorical data were presented as numbers and percentages, and numerical data were presented with mean, median, and minimum-maximum values. Statistical analyses were performed using SPSS version 21.0 for Windows (SPSS Inc., Chicago, IL).

## RESULTS

### Study Population

The study population consisted of 700 patients with the ratio of 532 females:168 males and ages ranging from 18 to 52 years.

### Follow-up

The average follow-up time was 16 months ranging from 12 to 24 months.

### Efficacy

All patients were effectively treated, as indicated by the study’s results. Clinical evaluation scores were recorded before the injections and at the 1-year follow-up. Prior to treatment, the average score on the GAIS was 1.6 out of 5, whereas after a year, it improved to 1.8 out of 5. Additionally, 82% of the patients (574 out of 700) had exceptional or great improvement, scoring 1 or 2 on the GAIS. The mean score for patient satisfaction was 4.4 out of 10 before the injection and 8.2 out of 10 after a year, with 85% of patients (595 out of 700) reporting high levels of satisfaction with a score of 8 or above. Further details can be found in Table 3.

**Table 3.** Patient Satisfaction Scores and GAIS Results Showing Two Independent Plastic Surgeons’ Objective Clinical Evaluation Scores After Injection

Patient Satisfaction Scores (0-10)		Global Aesthetic Improvement Scale (1-5)	
*0: Not satisfied, 10: Very satisfied*		*1: Best result, 5: Worst result*	
Before injection	1 year follow-up	Plastic Surgeon A	Plastic Surgeon B
Mean score: 4.4	Mean score: 8.4	Mean score: 1.6	Mean score: 1.8
Median: 5	Median: 8	Median: 2	Median: 2
Min-max: 2-6	Min-max: 6-9	Min-max: 1-3	Min-max: 1-3

### Duration

**Figure 2.**
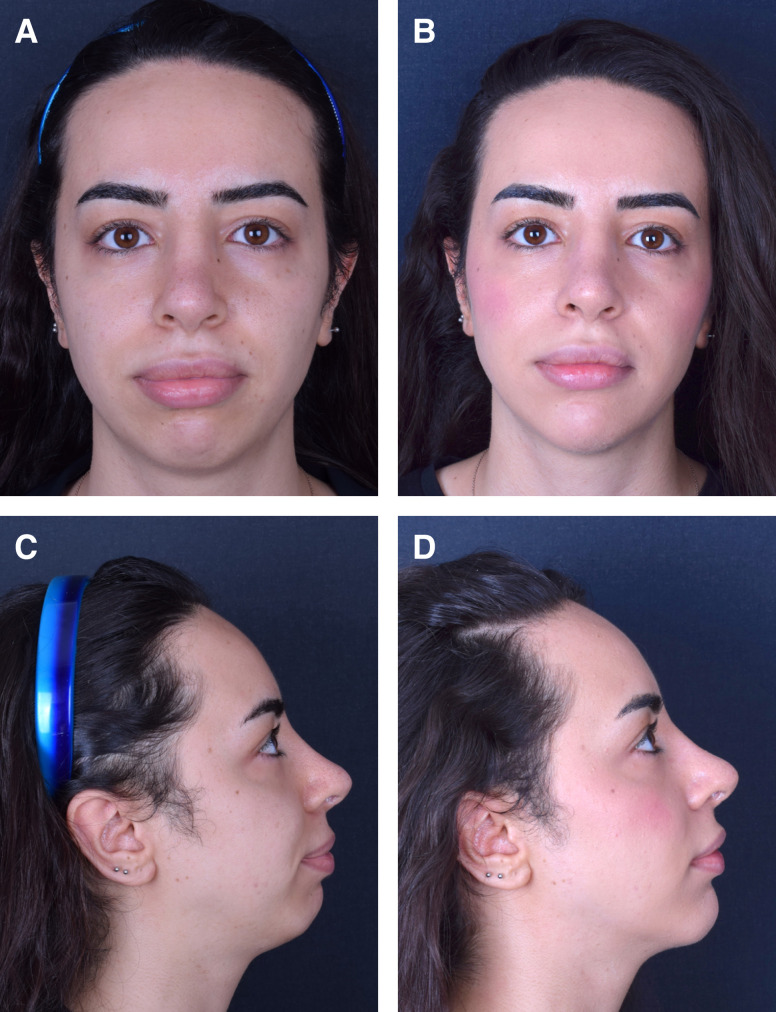
(A, C) Preprocedure and (B, D) 2 years postprocedure results of a 26-year-old female can be seen after 1.4 mL of 3.5% AG injection supraperiosteally to chin. Follow-up images showing the remaining clinical effects can be seen 2 years after the initial treatment.

At the 1-year follow-up, patients exhibited a minimum of 80% persistence of effect, irrespective of the treatment site. However, after 1.5 years, the outcome varied depending on several factors, such as age, depth of depression, location, and tissue thickness. Figures 2 through 6 showcase the results of patients who were treated with AG at our practice.

**Figure 3.**
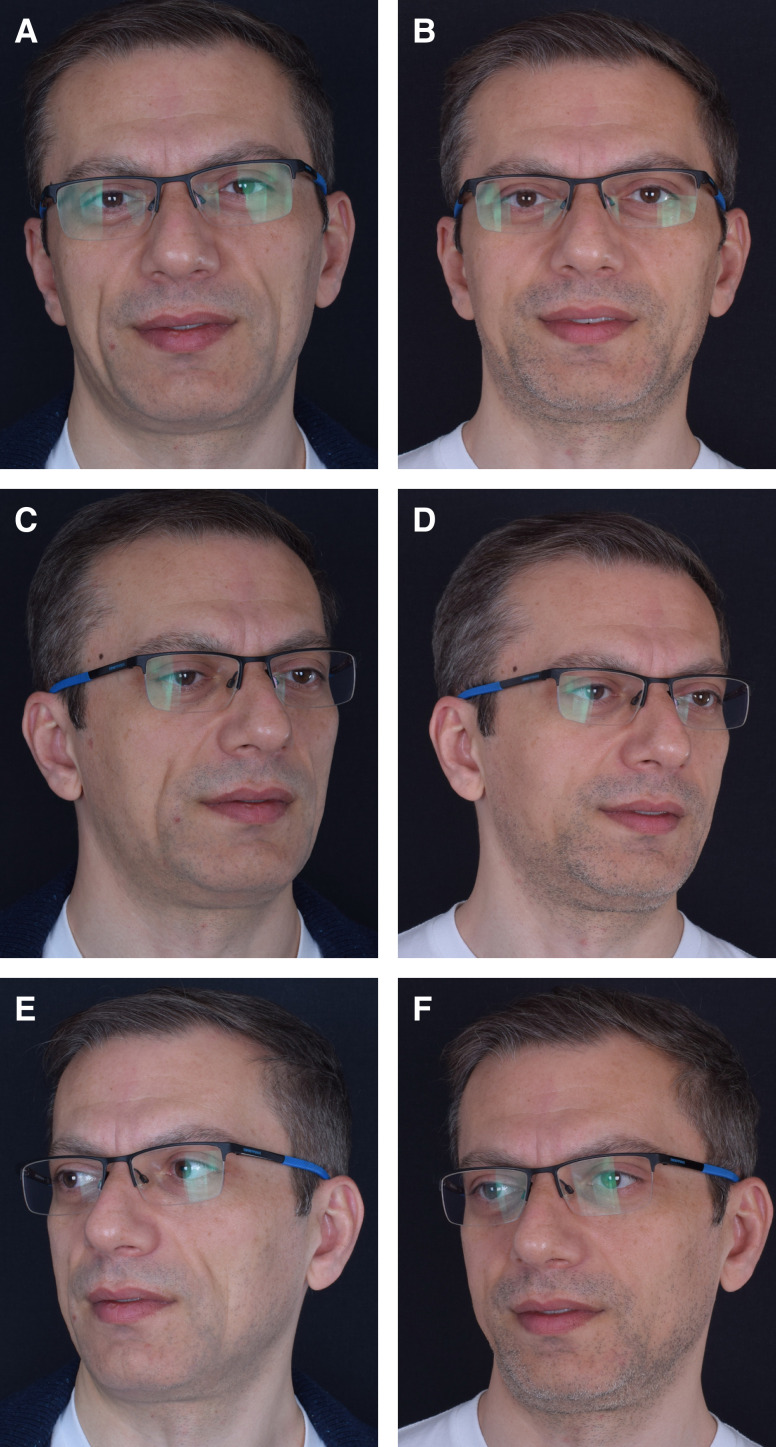
A 47-year-old male patient (A, C, E) prior to treatment of smile lines and (B, D, F) 12 months after injection of 3.5 mL of 3.5% AG to each side. The procedure was performed on subdermal plane using linear threading technique with a 23 G cannula.

**Figure 4.**
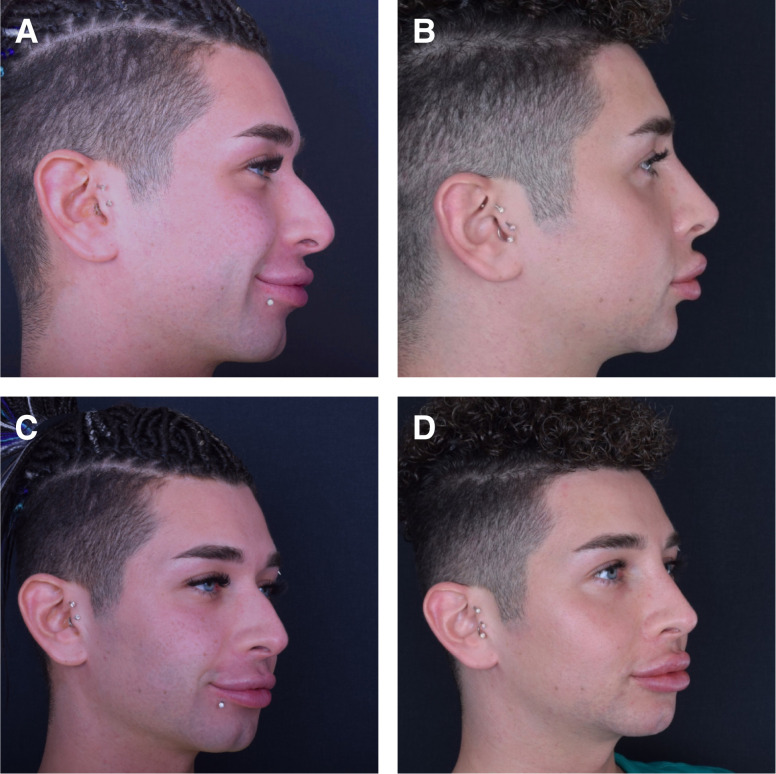
A 28-year-old male patient (A, C) prior to treatment and (B, D) 2 years after initial injection and 1-month touch-up treatment of 0.7 mL of %3.5 mL AG to radix and nasal spine each, combined with HA filler to tip and supratip area.

**Figure 5.**
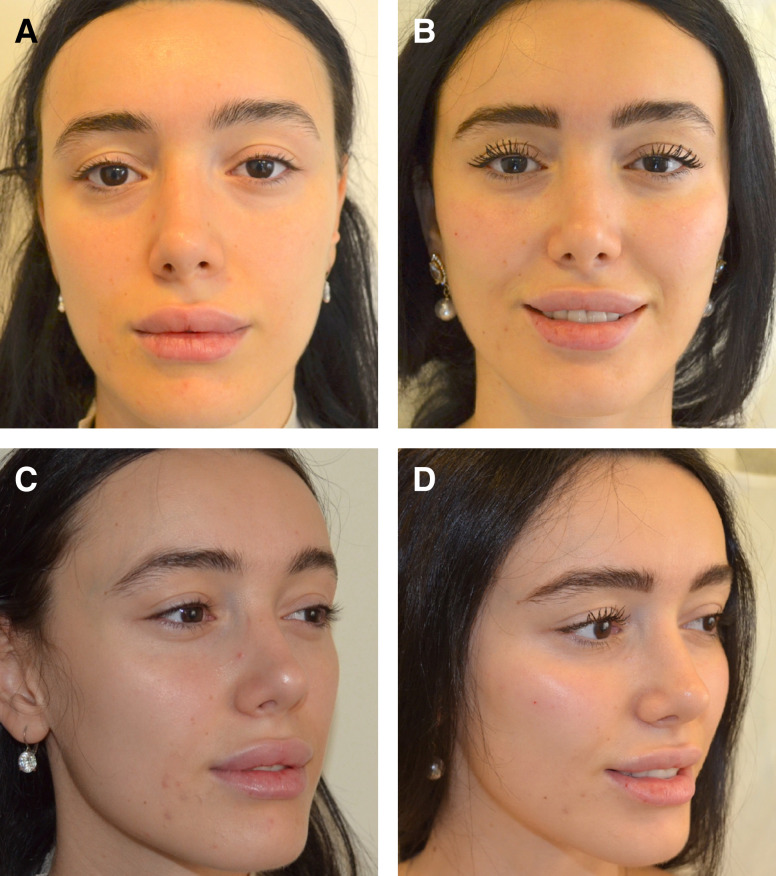
(A, C) Preprocedure and (B, D) 2 years postprocedure results of 22-year-old female patient can be seen after 1.4 mL of 3.5% AG injection to each zygoma on the supraperiosteal plane. A total of 2.8 mL of 3.5% AG filler was used in the procedure.

**Figure 6.**
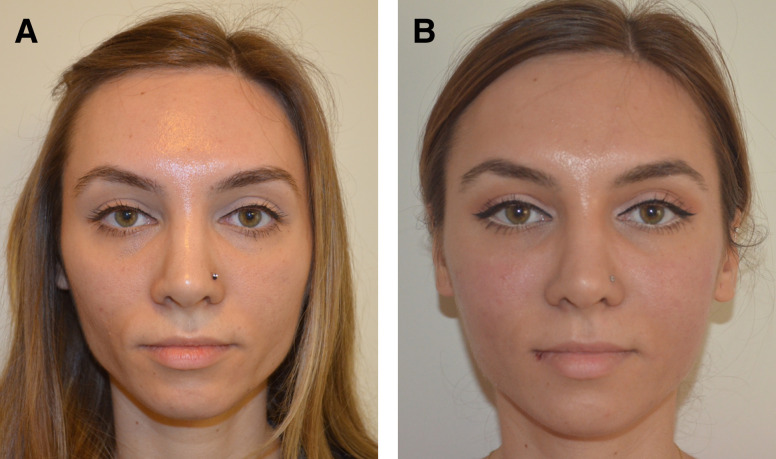
A 34-year-old female patient (A) prior to treatment of nasolabial area and smile lines and (B) 18 months after the injection of 2.1 mL of 2.5% AG to each side can be seen. The procedure was performed on subdermal plane using linear threading technique with a 23 G cannula.

### Safety

All patients had experienced mild erythema and edema at the injection site at the end of the procedures that resolved spontaneously within 24 h. Although some patients experienced mild discomfort during the injection, it was easily overcame by slower injections. Regarding long-term follow-ups, 1 chin augmentation patient had an infection localized to the area augmented. All of the patients had taken Cefuroxime 500 mg treatment, one of them was drained, all fully recovered without any sequela. A total of 5 lumps were identified during the follow-ups. All of them were invisible but palpable. Over time, lumps gradually disappeared along with the filler without any specific intervention. Lastly, 3 migration-displacement events were encountered. All migration cases were noticed during injection and corrected with adequate massage and molding immediately. No other serious adverse events or major complications, such as vascular injections, nodules, granulomas, or other complications were reported at any time point (Table 4).

**Table 4.** Complications of AG Dermal Filler Applications in Our Study

Complications	Chin	Temple	Zygoma	Nose	Nasolabial area, smile lines
Allergic and hypersensitivity reactions	—	—	—	—	—
Infection	1	—	—	—	—
Lumps	—	—	1	2	2
Nodule and granulomatous reactions	—	—	—	—	—
Migration, displacement	2	—	—	1	1

AG, agarose gel.

## DISCUSSION

Owing to its characteristics, AG is a biocompatible material that can be used in various preclinical and clinical applications. It has been used as a substrate for cell growth and microencapsulation and utilized in biocompatibility tests such as cytotoxity, mutagenesis, genotoxicity, and sensitivity.^12-20,27-32^

Hydrogels are a versatile material that can be employed for developing both 3D scaffolding and injectable systems. The synthesis of hydrogels can be tailored to impart specific geometric, physical, and chemical properties to meet the requirements of the target tissue. In this context, AG has been investigated for 3D tissue growth in bioengineering and as a polymer for tissue engineering applications.^15,33-35^ It has also been extensively researched for drug delivery applications, wherein it is utilized for controlled release of pharmacological substances, and as a carrier for cells and drugs.^34,36,37^

Agarose has a wide range of applications in tissue engineering, including its use as a temporary scaffold for growth factors and bony cells. It is also employed as a biocompatible substrate for bone-grafting procedures, and as a bone spacer for guided tissue regeneration in orthopedic, oral, and maxillofacial surgical procedures.^35,36,38-41^

It is also used in different applications in various areas: in microbiology as culture media, in pharmaceuticals and phytotherapy applications as thickeners, as a laxative, a defense for the mucous membranes of the gastrointestinal tract.^25^ Agar has been employed in the food industry as a stabilizer, thickener, humectant, surface finisher, and flavoring agent. It is considered a safe ingredient and complies with the specifications of the Food Chemicals Codex.^42^

AG can be utilized as an alternative treatment option for several functional disorders in the head and neck region, as an example of its clinical applications. It has been used for the management of periprosthetic leaks after total laryngectomy and for the prevention of recurrent aspiration pneumonia. In addition, promising functional indications with long-term outcomes have been observed in the treatment of unilateral vocal cord paralysis using AG.^43^

As the face and neck age, the loss of bone and fat volume, formation of wrinkles, and reduction in elastin and collagen can manifest. Moreover, congenital or acquired deformities in the facial features, nose, ears, lips, and scars may require aesthetic intervention for correction. In these cases, AG can be used as the primary choice due to its reshaping and molding properties.

Nasal dermal filler applications are used for dorsal augmentation or to cover defects, such as irregularities or deviations for camouflage purposes.^1-3^ Considering its structural properties, nonhydrophilic AG can be used in the supraperiosteal plane in the radix and nasal spine without causing undesirable swelling.^44^ Linear retrograde injection is the recommended injection technique in AG applications, as intradermal and big bolus injections can cause palpability and lump formation. However, serial small bolus injection has been found to give good results if it is over the supraperiosteum or subSMAS. Given the persistency of the fillers, according to the studies with a 2-year follow-up, 3.5% AG fillers indicated for radix and nasal spine could last up to 18 months which is comparable to other fillers currently on the market.^4,44^

As individual’s age deepening of the nasolabial folds is a common occurrence. To address this issue, AG can be injected in Ristow’s space, below the ala of the nose and the elevator muscle of the lower lip, using higher density preparations such as AG 2.5% or 3.5%. However, care must be taken during injection to avoid inadvertent spread of the product from the fibrous lip subcutis to the softer cheek subcutis, which can result in unwanted nasolabial fullness. To prevent this, lateral compression of the cheek can be applied during injection. This technique can also help to prevent the injectable from spreading intraarterially.^11,43^

Mandibular and chin volumization can augment, refine, and improve the definition of the entire face. AG 2.5% or 3.5% can be used supraperiosteally using small boluses and linear retrograde technique.^43^

The zygomatic region can be treated with AG 2.5% or 3.5% supraperiosteally. It is important to inject the filler slowly and to mold it carefully after injection to ensure proper shaping.^43^

In cases of depression, the temple can be successfully treated with AG 2.5% or 3.5%. Placing AG deep in the temporalis muscle can help avoid the superficial temporal artery, which is a major blood vessel located on the surface of the temple. Injection can be performed supraperiosteally using big bolus injection into the area defined.^43^

In a study, a concentration of 2.5% wt AG was proposed for lip augmentation in 62 patients, resulting in immediate clinical improvement that lasted for 5 months.^19^ We have been used AG and HA fillers in a hybrid combination for nonsurgical rhinoplasty procedures to 32 patients in 2 years period which yielded very good and durable results.^44^ In another study, It has been shown that 3.5% AG can last up to 12 months or even longer to 16 months.^4^ Regarding their comparable durations, 3 to 5 months of duration can be expected for 1% AG. Four to 8 months are typical for 1.5% and 2.5% AG, while longer durations of up to 12 to 18 months can be seen with 3.5% AG fillers.^4,19,44^

Immediately after AG injections, some erythema, swelling and tenderness may occur at the injection site. These findings are temporary and will resolve within a few hours or a few days.^45^

Filler injections can result in complications such as vessel occlusions, leading to bleaching, pain, ischemia, and tissue necrosis.^45-47^ Precautions should be taken to minimize the risk of embolization, such as lifting the skin and applying pressure to important vessel origins during needle insertion, using linear threading technique and aspirating for 5 s before the insertion.^11,43,44^ It is also crucial to be aware of the facial anatomy and key vessels, such as the angular artery around the nasolabial fold, and to respect the recommended tissue layer around the zygomatic region to avoid key vessels.^43^

So far, there have been no cases of intravascular injection complications associated with the use of AG fillers reported in the literature. In the event of any complications, hyaluronidase can be added to the treatment procedure in particulate fillers, since noncross-linked HA is present in the preparations at concentrations of 2.5% and 3.5%.^44,48^

We kindly do not recommend injecting AG intradermally in a bolus fashion to avoid palpability of macrophage conglomerates constitute lump formation. Posttreatment massage is important and should be considered to decrease the risk of palpability, especially when injecting to thin skin areas such as lips or nose.^19,22,44^

Complications and major side effects such as abscesses, inflammatory nodules and granulomas may occur after injectables. However, no such case has yet been reported regarding AG injections.^43,44^ Lumps may also form due to the low migration effect of AG when big bolus injections are administered. Therefore, the application method is of great importance.

Our experience with AG showed it to be a safe and versatile injectable soft-tissue filler. To our knowledge, this is the largest clinical study to date on AG in facial aesthetic procedures. The limitation of the presented study is that it is not a double-blind comparative study. We are pleased with the low incidence of adverse events with high patient satisfaction rates. In an earlier study we performed, 90.6% of 32 patients reported very satisfied with their results following the treatment.^44^

## CONCLUSIONS

Beginning with aesthetic indications, reaching functional disorder treatments, the indications of AG have expanded considerably thanks to its unique features over the years. The contouring ability makes AG a good lifting and shaping tool. Knowing how to use it, which danger zones to avoid, and to manage potential complications paves the way for optimal clinical outcomes. In conclusion, AG dermal filler is suitable for various facial augmentation and contouring applications, as it is safe and has long-lasting favorable cosmetic efficacy.

## Supplemental Material

This article contains supplemental material located online at www.asjopenforum.com.

## Supplementary Material

ojad051_Supplementary_Data
